# P-284. Evaluation of Patients Living with HIV Before and After Implementing a Reconnecting to Care Intervention for Patients who have Fallen out of Care

**DOI:** 10.1093/ofid/ofaf695.505

**Published:** 2026-01-11

**Authors:** Thomas Ludden, Jeremy Thomas, Michael Leonard, Eboni Sanders, Hazel Tapp

**Affiliations:** Atrium Health, Charlotte, North Carolina; Atrium Health, Charlotte, North Carolina; Atrium Health, Charlotte, North Carolina; Atrium Health, Charlotte, North Carolina; Atrium Health, Charlotte, North Carolina

## Abstract

**Background:**

While the benefits of reconnecting patients living with HIV and AIDS (PLWHA) to care are well-documented, more than one-third of these patients who know their serostatus are not connected/reconnected to medical care. PLWHA fall out of medical care for a variety of reasons. These may include, but are not limited to, personal barriers such as unemployment, lack of health insurance, substance use, mental health issues, lack of transportation, and/or homelessness. 87% of new HIV infections are transmitted from people who don’t know they have HIV or are not in care. This reconnecting to care intervention was designed to increase the number of PLWHA back into medical care.

**Methods:**

A pre-intervention cohort of patients who had not attended a medical appointment for at least six months was identified from April 2023 to March 2024. Between April 2024 and March 2025, a post-intervention cohort who had not attended a medical appointment for more than 6 months and did not have an appointment on record was identified as having fallen out of care. The post-intervention cohort was contacted, given an appointment for medical care, and completed that appointment. Demographic statistics of the pre- and post-intervention cohorts were compared using t-test statistics by gender and race.

**Results:**

Of the 185 post-intervention cohort contacted, 138 completed medical appointments versus 91 identified in the pre-intervention cohort who completed medical appointments (50% increase). The comparison analysis showed a difference in gender (pre-intervention 62.7% male vs post-intervention 81.5% male, P< 0.0001). For race, there was no statistically significant difference for either White/Caucasian (pre-intervention 26.4% vs post-intervention 23.7%, P=0.6920) or Black/African American (pre-intervention 68.1% vs. post-intervention 69.8%, P=0.801).

**Conclusion:**

The reconnecting to care intervention increased the number of patients in medical care by over 50%. While the race characteristics of the two groups did not differ, male patients were represented in higher proportions in the post-intervention group.

**Disclosures:**

Thomas Ludden, PhD, Gilead Sciences: Grant/Research Support Jeremy Thomas, MSW, Gilead Sciences: Grant/Research Support Michael Leonard, MD, Viiv Healthcare: Grant/Research Support Eboni Sanders, MA, Gilead Sciences: Grant/Research Support Hazel Tapp, PhD, Gilead Sciences: Grant/Research Support

